# 2198. Assessing Strengths and Opportunities of Antibiotic Stewardship Programs in Philadelphia Skilled Nursing Facilities

**DOI:** 10.1093/ofid/ofad500.1820

**Published:** 2023-11-27

**Authors:** Mariah Menanno, Jane Gould, Tiina Peritz, Elizabeth Welsh, Deborah Heisey, Jerry Jacob

**Affiliations:** Philadelphia Department of Public Health, Philadelphia, Pennsylvania; 1Philadelphia Department of Public Health, Philadelphia, PA, USA, Philadelphia, Pennsylvania; Philadelphia Department of Public Health, Philadelphia, Pennsylvania; Penn Medicine, Philadelphia, Pennsylvania; Penn Medicine, Philadelphia, Pennsylvania; University of Pennsylvania, Philadelphia, Pennsylvania

## Abstract

**Background:**

Skilled nursing facilities (SNFs) have significant opportunity to improve antibiotic use and reduce multidrug-resistant organism transmission. In the summer of 2022, the Southeast Pennsylvania Long-Term Care Resiliency, Infrastructure Supports, and Empowerment (LTC-RISE) team and the Philadelphia Department of Public Health (PDPH) distributed a survey to all Philadelphia SNFs to understand strengths and opportunities for improvement of antibiotic stewardship programs (ASPs).

**Methods:**

A REDCap survey was distributed via phone or video call to 47 SNFs addressing all elements in the CDC's Checklist for Core Elements of Antibiotic Stewardship in Nursing Homes. SNFs self-reported survey responses. Survey results were used to provide individualized feedback highlighting SNF performance against peers, and to implement broad-based interventions to strengthen ASPs.

**Results:**

Thirty-eight SNFs (81%) responded to the survey. All 7 ASP core elements were reported present by 55% of respondents. Only one SNF did not have any core elements present. The elements most commonly reported present and absent were tracking measures of antibiotic use (97%), and monitoring outcomes of antibiotic use (79%), respectively. The ASP lead(s) most commonly identified was the Infection Preventionist (66%), Director/Assistant Director of Nursing (55%), or Medical Director (37%). Infection-specific interventions and resident educational materials were provided in only 42% and 34% of SNFs, respectively. Customized ASP reports were sent to all participating SNFs, with feedback obtained at 3 months after distribution. Survey results were used to create an educational webinar and UTI-focused resource package.

**Conclusion:**

A majority of Philadelphia SNFs reported having ASPs with all CDC core elements in place, and almost all facilities tracked at least one measure of antibiotic use. Opportunities for improvement included tracking outcomes of antibiotic use, providing infection-specific interventions, and providing educational materials to residents. Insights provided from the survey helped to guide individualized and broad-based ASP interventions across facilities.

**Disclosures:**

**All Authors**: No reported disclosures

